# Automatic Plugger

**Published:** 1866-06

**Authors:** 


### AUTOMATIC PLUGGER.

We have had upon our case for several months one of these beautiful little instruments made by Snow & Lewis, and have occasionally brought it into requisition.

We regard it as one of the best instruments of the kind that have been constructed. We presume that one who had not been spoiled by the living assistant, would find this to be one of the most valuable instruments; and especially after having become accustomed to its use and forming an attachment for it. Very much, in the use cf anything, depends upon the good opinion that may be formed of it. We oftentimes see persons using instruments, with great facility, and even accomplishing fine results —that seem to lack almost every element of adaptability and fitness.

This instrument, we are glad to say, seems at once to be just the thing for accomplishing that for which it is intended; and no operator who does not have an assistant constantly at his side, should be without one of these instruments. They are for sale at the Dental Depots.

